# Oxidized Phospholipids in Healthy and Diseased Lung Endothelium

**DOI:** 10.3390/cells9040981

**Published:** 2020-04-15

**Authors:** Pratap Karki, Konstantin G. Birukov

**Affiliations:** 1 Division of Pulmonary and Critical Care Medicine, Department of Medicine, University of Maryland School of Medicine, Baltimore, MD 21201, USA; pkarki@som.umaryland.edu; 2 Department of Anesthesiology, University of Maryland School of Medicine, Baltimore, MD 21201, USA

**Keywords:** oxidized phospholipids, lung injury, inflammation, endothelial barrier, phospholipidomics, aging

## Abstract

Circulating and cell membrane phospholipids undergo oxidation caused by enzymatic and non-enzymatic mechanisms. As a result, a diverse group of bioactive oxidized phospholipids generated in these conditions have both beneficial and harmful effects on the human body. Increased production of oxidized phospholipid products with deleterious effects is linked to the pathogenesis of various cardiopulmonary disorders such as atherosclerosis, thrombosis, acute lung injury (ALI), and inflammation. It has been determined that the contrasting biological effects of lipid oxidation products are governed by their structural variations. For example, full-length products of 1-palmitoyl-2-arachidonoyl-sn-glycero-3-phosphorylcholine oxidation (OxPAPC) have prominent endothelial barrier protective and anti-inflammatory activities while most of the truncated oxidized phospholipids induce vascular leak and exacerbate inflammation. The extensive studies from our group and other groups have demonstrated a strong potential of OxPAPC in mitigating a wide range of agonist-induced lung injuries and inflammation in pulmonary endothelial cell culture and rodent models of ALI. Concurrently, elevated levels of truncated oxidized phospholipids are present in aged mice lungs that potentiate the inflammatory agents-induced lung injury. On the other hand, increased levels of full length OxPAPC products accelerate ALI recovery by facilitating production of anti-inflammatory lipid mediator, lipoxin A4, and other molecules with anti-inflammatory properties. These findings suggest that OxPAPC-assisted lipid program switch may be a promising therapeutic strategy for treatment of acute inflammatory syndromes. In this review, we will summarize the vascular-protective and deleterious aspects of oxidized phospholipids and discuss their therapeutic potential including engineering of stable analogs of oxidized phospholipids with improved anti-inflammatory and barrier-protective properties.

## 1. Introduction

Lipid mediators are integral components of cell membranes providing the structural integrity and acting as a source of energy production. However chemical modifications, in particular, oxidation, generates a wide variety of bioactive oxidized phospholipids (OxPLs) that have profound effects on intracellular signaling and modulation of cell functions [1]. The generation of OxPLs may be a result of specific enzymatic reactions or non-enzymatic oxidation of blood-bound or cell membrane phospholipids by reactive oxygen species (ROS) [2]. In this regard, OxPLs differ from other lipid mediators such as prostaglandins, which are exclusively produced by enzymatic oxidation. Polyunsaturated fatty acids (PUFA) at the *sn*-2 position of phospholipids represent the principal site for enzymatic and non-enzymatic oxidation [3,4]. The major groups of enzymes involved in the oxidation of phospholipids are lipoxygenases (LOX) and cyclooxygenases [1,3]. Increased production and accumulation of OxPLs has been well recognized as a pathological feature of numerous diseases including atherosclerosis, lung inflammation, apoptosis, multiple sclerosis, traumatic brain injury, asthma, and acute respiratory distress syndrome (ARDS) [5,6,7,8,9,10,11,12,13]. However, the emerging evidence suggests that certain full-length OxPLs exhibit pro-survival and anti-inflammatory effects. Beneficial properties of OxPLs have been extensively studied using oxidation products of 1-palmitoyl-2-arachidonoyl-*sn*-glycero-3-phosphorylcholine (PAPC), a major constituent of the outer leaflet of cell membrane phospholipid bilayer. Studies by our group and other groups established oxidized PAPC products (OxPAPC) as key bioactive products that protect against vascular leak and inflammation induced by diverse stimuli [3,14,15]. Herein, we will summarize the current knowledge on the dual effects of OxPLs with a focus on lung endothelium and discuss signaling mechanisms involved in these processes.

## 2. OxPLs in Health and Disease

The involvement of OxPLs in the pathogenesis of cardiopulmonary diseases was initially suggested by the findings that the levels of lipid peroxidation products, isoprostanes, were elevated in patients with atherosclerosis, acute lung injury (ALI), ARDS, asthma, pulmonary hypertension, and cystic fibrosis [16,17,18,19,20]. These studies showed that isoprostanes may serve as in vivo markers of oxidative stress. OxPLs were also shown to act on endothelial cells (EC, focus of this review). EC lining the luminal surface of blood vessels form a highly selective semipermeable barrier between the blood and underlying tissues to control the passage of fluid, macromolecules, and immune cells. EC stimulated with increased OxPLs doses relevant to hyperlipidemic and atherosclerotic conditions show enhanced adhesion of monocytes, upregulation of several pro-inflammatory genes, and increased secretion of cytokines and chemokines including interleukin (IL)-8 and monocyte chemotactic protein-1 (MCP-1) [21,22,23,24]. Similarly, a role of OxPLs in EC-mediated coagulation has been demonstrated by OxPL-induced increase in expression of pro-thrombotic tissue factor (TF) by EC and decrease in anticoagulant activity of tissue factor pathway inhibitor (TFPI) [25,26]. Additionally, OxPLs are involved in the generation of excessive ROS by EC that contributes to lung inflammation and injury. EC exposure to high-dose OxPLs caused depletion of intracellular antioxidant glutathione caused by increased superoxide production via OxPLs-induced upregulation of nicotinamide adenine dinucleotide phosphate (NADPH) oxidase and endothelial nitric oxide synthase (eNOS), thus leading to oxidative stress [27,28,29,30].

Interestingly, in addition to the aforementioned roles in chronic vascular inflammation, coagulation, and oxidative stress, OxPLs have been shown to exhibit anti-inflammatory and other beneficial cellular effects in the context of acute innate immune response. The seminal study by Bochkov et al. showed that OxPLs inhibited lipopolysaccharide (LPS)-induced inflammation in cultured EC and protected mice from LPS-induced lethality [31]. Consistent with these findings, another study showed that OxPLs inhibited LPS and 2′-deoxyribo(cytidine-phosphate-guanosine) (CpG)-induced production of proinflammatory cytokine tumor necrosis factor-α (TNF-α) in mice [32]. Concurrently, the study from our group revealed that OxPLs enhance basal endothelial barrier function and restore thrombin-induced EC hyperpermeability [33]. We also verified the barrier protective and anti-inflammatory modalities of OxPLs in murine model of ALI, demonstrating that OxPLs offer protection against LPS-induced vascular leak and inflammation in rats [34]. Follow-up studies by several groups substantiated anti-inflammatory role of OxPLs in animal models of acute septic inflammation [31,33,35,36,37]. The potential of OxPLs to inhibit endothelial barrier disruption and lung inflammation induced by a wide variety of agonists ranging from bacterial pathogens, inflammatory, and edemagenic agents to mechanical forces will be discussed below.

## 3. Structural Heterogeneity Determines the Differential Effects of OxPLs

**Figure 1:**
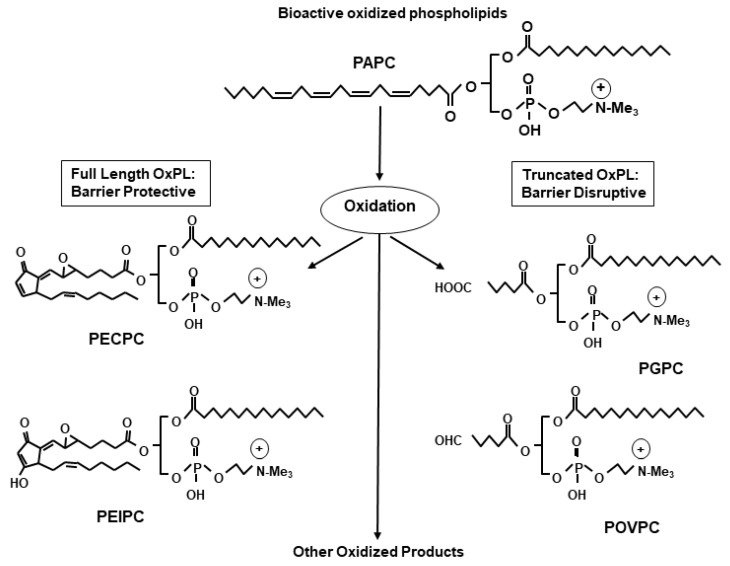
Generation of full-length and truncated oxidized phospholipids from the oxidation of membrane phospholipid: A wide varieties of oxidized phospholipids (OxPLs) are generated from the oxidation of phospholipid, and among these, full length OxPLs have endothelial barrier protective properties while truncated ones induce barrier disruption.

Non-enzymatic oxidation of phospholipids produces a heterogenous mixture of bioactive OxPL species. This phenomenon is best exemplified during the oxidation of a major membrane phospholipid PAPC resulting in generation of families of full-length as well as fragmented oxidized products (Figure 1). The full-chain OxPAPC products contain the same number of carbon atoms in the oxidized arachidonic fatty acid chain as in their precursor. Examples include 1-palmitoyl-2-(5, 6-epoxyisoprstane E2)-sn-glycero-3-phosphatidyl choline (PEIPC), and 1-palmitoyl-2-(5,6 epoxycyclopentenone) *sn*-glycero-3-phsphocholine (5,6-PECPC). Similarly, truncated products of PAPC oxidation are represented by lysophosphatidylcholine (lyso-PC), 1-palmitoyl-2-(5-oxovaleroyl)-sn-glycero-phosphatidylchonine (POVPC), 1-palmitoyl-2glutaroyl *sn*-glycero-phosphocholine (PGPC), 5-keto-6-octendioic acid ether of 2-lysophosphocholine (KOdiA-PC), and other products [1]. With such a variety of OxPL compounds generated from the oxidation of a single parental phospholipid, it is not surprising that the structural differences dictate contrasting biological effects of OxPLs. Indeed, a full-length OxPAPC product, PEIPC, demonstrated potent protective effects on cultured human lung EC monolayers, while truncated OxPAPC products such as PGPC, POVPC, and lyso-PC caused EC barrier disruption [38]. Prevalence of barrier disruptive activities by truncated oxidation products present in total OxPAPC pool explains EC barrier function caused by higher OxPAPC doses, while only low doses of OxPAPC strongly enhanced EC barrier and abolished EC permeability caused by barrier disruptive agonists [33,37,39]. These dose-dependent differential effects were largely due to the activation of different signaling pathways which will be discussed below. Nevertheless, these findings justify the clinical scenario where excessive accumulation of OxPLs causes deleterious effects on vasculature. Overall, there is now a general consensus in the field that the biological effects of OxPLs vary depending on their structure, intracellular concentration, and cellular context.

Other groups of lipid oxidation products such as hydroxyoctadecadienoic acids (HODEs) and Hydroxyeicosatetraenoic acids (HETEs) are also known to play important physiological roles and may modulate endothelial function. Both HODEs and HETEs act as ligands for peroxisome proliferator-activated receptor gamma (PPARγ) and thus regulate various cellular functions [40]. ECs synthesize 9- and 13-HODEs that are formed from the oxidation of linoleic acid and are the most abundant low-density lipoprotein in atherosclerotic lesions [41]. Furthermore, 13-hydroperoxyoctadecadienoic acid (13-HPODE), which is the predominant product of 15-LOX-mediated oxidation and later gets reduced into 13-HODE, causes apoptosis and disrupts barrier integrity in EC [42]. However, a recent study has shown that 20-HODE-caused disruption of endothelial barrier is not mediated by oxidative stress or apoptosis [43]. 13(S)-HODE causes airway epithelial injury via mitochondrial dysfunction, and in the transient receptor potential cation channel, vanilloid-type 1 (TRPV1)-dependent manner and levels of 13(S)-HODE are increased in asthma [44]. Similarly, 12/15-LOX-mediated generation of 12-HETE is a key contributor in LPS or acid-induced ALI in mice [45]. 12- and 15-HETE increase endothelial permeability in retinal EC via NADPH-dependent ROS production [46]. Similarly, 12(S)-HETE combined with high glucose induces endothelial barrier disruption by altering the phosphorylation levels of VE-cadherin and b-catenin and by reducing their interactions in human umbilical vein EC [47]. 12(S)-HETE and high glucose-induced endothelial barrier disruption was accompanied by increased endothelial inflammation with increased phosphorylation of nuclear factor kappa-light-chain-enhancer of activated B cells (NF-κB) and inhibitor of NF-κB (IkBα) along with the increased expression of EC adhesion molecules intercellular adhesion molecule-1 (ICAM-1) and vascular cell adhesion molecule-1 (VCAM-1) [47]. A number of studies have also reported the role of 15-HETE in angiogenesis of lung EC [48,49] and in pulmonary vascular remodeling leading to pulmonary hypertension [50,51].

## 4. OxPLs in Inflammation: Role and Mechanisms

Accumulation of OxPLs occurs in inflamed tissues, as evidenced by increased levels of truncated OxPAPC products in atherosclerotic lesions [8], inflamed lungs [52], and the brain [5]. OxPLs are initially produced from lipid peroxidation during oxidative stress developing as a part of host–pathogen response. Once accumulated in inflamed areas, truncated OxPLs propagate inflammation by various mechanisms. Among these, OxPLs induce monocytes binding to EC initiating inflammation [22], thus playing a role in the formation of atherosclerotic plaque. EC-monocyte interaction is facilitated by OxPLs-induced secretion of monocyte-specific chemoattractant MCP-1. Furthermore, OxPLs also increase the deposition of connecting segment 1 (CS1) fibronectin on EC surface that binds to integrin α4β1 receptor on monocytes resulting in firm adherence of monocyte on EC [53,54]. Additionally, a role of LOX in OxPLs-induced monocytes adhesion on EC has been suggested based on findings that LOX inhibitor represses POVPC-induced monocyte binding to EC [55]. The mechanism of OxPLs-induced monocyte–EC interaction is unique in a sense that OxPLs do not upregulate the expression of EC surface adhesion molecules ICAM-1, VCAM-1, and E-selectin as in the case of other common proinflammatory agonists such as LPS or TNF-α [56]. Rather, OxPLs upregulate the expression of P-selectin, which facilitates the selective adhesion of monocyte on EC. In regard to the role of OxPLs in modulating the function of monocytes in inflammation, recent studies have shown that OxLDL increases mTOR-dependent ROS formation, inflammatory cytokine production and foam cell formation by mTOR-dependent and epigenetic pathways [57,58].

Another mechanism which OxPLs employ to initiate and propagate inflammation is increased production of an array of proinflammatory cytokines including IL-6, IL-8, MCP-1, MIP-1α, MIP-1β, CXCL3 GROα, and GROβ by EC and macrophages [22,59,60]. The mechanisms of OxPLs-induced generation of inflammatory cytokines and chemokines may substantially differ from those involved in typical inflammatory agents or bacterial and viral pathogens. For instance, the kinetics of IL-8 transcription by OxPLs in pulmonary EC varies from that of TNF-α. Both OxPLs and TNF-α induce IL-8 mRNA as early as 30 minutes, but the highest levels are seen between 4–8 hours in the case of OxPLs compared to 2 hours with TNF-α [61]. Notably, TNF-α-induced IL-8 mRNA levels drop to baseline after 6 hours, whereas they last up to 18 hours in OxPLs stimulated groups. Interestingly, in contrast to the common NF-κB pathway activated by most inflammatory agonists, OxPLs utilize different signaling cascades in the production of these inflammation mediatory cytokines. Other studies have suggested a role for peroxisome-activated receptor alpha [22], c-Src/signal transducers and activators of transcription 3 (STAT3) [62], eNOS-mediated activation of sterol regulatory element-binding protein (SREBP) [27,63], and unfolded protein response following endoplasmic stress [64,65] in OxPLs-induced inflammatory responses. Furthermore, recent studies have shown that OxPLs activate nod-like receptor protein-3 (NLRP3) inflammasome, as demonstrated by POVPC-stimulated activation of procaspase-1, leading to the generation of mature IL-1β and IL-8 in macrophages [66]. That study suggested a strong involvement of the NLRP3 inflammasome pathway by showing a lack of IL-1β and IL-8 generation in NLRP3-deficient mice, which is normally activated in air pouch model. OxPLs have been also shown to activate inflammasome by direct binding to caspase-11 and releasing IL-1β in dendritic cells [67]. The important caveat of these studies is unclear precise composition of OxPLs preparation used in inflammasome experiments.

Accumulating evidence suggests that, in various pathological states, OxPLs can act as danger-associated molecular patters (DAMPs) by activating pattern-recognition receptors (PRRs) and by contributing to chronic inflammation. It is likely that OxPLs formed as “self-modified” molecules following the oxidative modifications of phospholipids structurally resemble DAMPs [68]. A study by Imai et al. showed that OxPLs generated in lungs of mice infected with bacterial or viral pathogens stimulate Toll-like receptor 4 (TLR4) to induce lung injury [69], although precise composition of OxPLs was not determined. Another study reported that *tlr4* knockout mice are resistant to influenza-induced lung injuries and lethality, and this TLR4 inhibition-dependent protective effects is mimicked by LPS competitive antagonist eritoran [70]. These findings were consistent with an earlier study suggesting the role of TLR4 in OxPLs-induced IL-8 transcription [71]. However, the role of TLR4 in influenza-induced lethality has been challenged by other study [72]. A more recent study has shown that hydroxyl radical-produced OxPLs act as TLR4 ligands and exacerbate cellular senescence, inflammation, apoptosis, and fibrosis [73]. Nitrogen mustard-induced accumulation of pro-inflammatory OxPLs in lung macrophages and epithelial cells are suggested to contribute to the development of pulmonary fibrosis [74]. As opposed to the aforementioned role of TLR4, other studies have suggested that TLR2 mediates OxPLs-induced inflammation [75]. In addition to TLRs, OxPLs are also recognized by other several receptors, including scavenger receptors such as CD36 [76,77], and soluble PRRs such as C-reactive protein [7] which may play a role in mediating the inflammatory effects by OxPLs. It is intriguing that most of the above-described receptors are equally important and involved in anti-inflammatory effects by OxPLs.

Coagulation is a pathological phenomenon closely associated with inflammation, and like other many inflammatory agents, OxPLs stimulate the healthy endothelium to a procoagulant or thrombotic phenotype by modulating the expression of major proteins involved in these cascades. Studies have shown that OxPLs stimulate the activity and induce the expression of procoagulant protein TF on EC surface while reducing the activity of anticoagulant protein TFPI [25,26]. OxPL-induced stimulation of TF is mediated by the activation of extracellular signal related kinase (ERK) 1/2, early growth response factor 1 (EGR1), and increase in Ca^2+^ release with enhanced binding of nuclear factor of activated T cells (NFAT) [25]. Similarly, direct association of OxPLs with carboxy-terminal basic region of TFPI inhibits its activity [26]. OxPLs also cause the transcriptional repression of another anticoagulant glycoprotein thrombomodulin in vascular EC [78].

## 5. Anti-Inflammatory Effects of OxPLs and Involved Mechanisms

A large number of studies in the recent years have provided compelling evidences that OxPLs exert inflammatory and cytoprotective effects, making these molecules attractive potential therapeutic targets. The initial studies showed that OxPAPC is a potent inhibitor of LPS-induced inflammation in various cell types including EC and macrophages as well as in mice with its ability to interfere with TLRs signaling [31,32,53,79]. The anti-inflammatory effects of OxPLs were specific against LPS since they failed to inhibit the upregulation of inflammatory genes induced by TNF-α or IL-1β [31]. More importantly, OxPLs were equally effective in inhibiting inflammation in mice and protected LPS-injected animals from endotoxin shock-caused lethality. It is considered that blocking of TLR4 activation due to the direct binding of OxPLs to TLR4 activating proteins LPS-binding protein, CD14, and MD-2 is responsible for complete inhibition of LPS-induced inflammation [31,80,81]. These studies also identified that, besides TLR4, the target of anti-inflammatory actions of OxPLs is TLR2 since both of these TLR subtypes require CD14 for their optimal activation [79,80,82,83]. Later, Walton et al. proposed a different mechanism of OxPL-induced blunting of LPS signaling which involves the alteration of caveolae distribution and activation of neutral sphingomyelinase [84]. Moreover, the lecinoxoides family of OxPL synthetic analogs VB-201 and VB-703 are shown to inhibit central nervous system inflammation and liver fibrosis as well as inflammation [85,86]. OxPL preparations have been shown to modulate inflammatory responses of monocytes and myeloid dendritic cells by inhibiting inflammatory cytokines TNF-α and IL-1β production by these peripheral blood cells in response to LPS [87].

**Figure 2:**
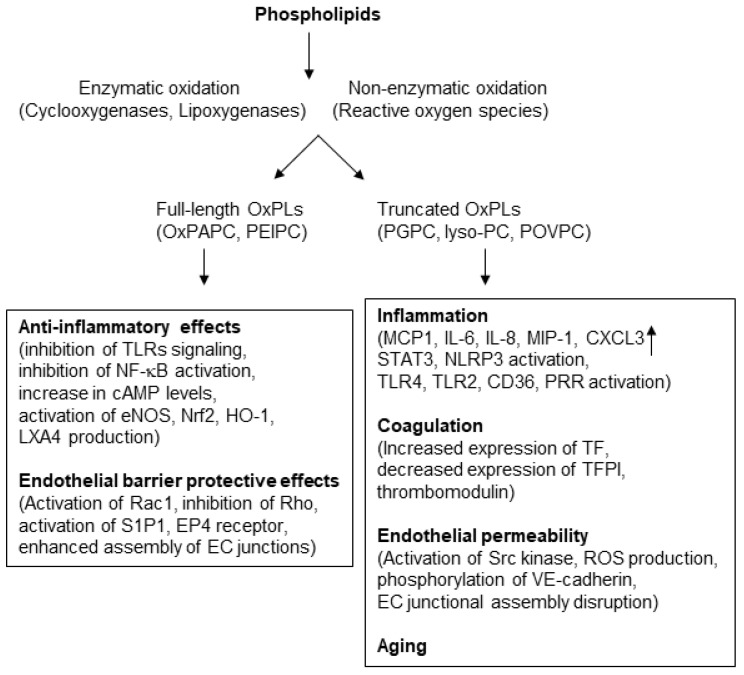
Diverse cellular functions of full-length and truncated oxidized phospholipids: Full-length OxPLs exert anti-inflammatory effects by various mechanisms including the inhibition of Toll-like receptor (TLR)-derived signaling pathways and activation of antioxidant molecules such as Nrf2. These OxPLs also enhance endothelial barrier function by upregulation of Rac-mediated cytoskeletal remodeling. In turn, truncated OxPLs cause inflammation by the activation of TLRs, induce coagulation, and increase endothelial permeability.

Various intracellular signaling pathways are implicated in mediating the anti-inflammatory effects of OxPLs. For example, the study by Ma et al. showed that OxPLs-induced inhibition of LPS or CpG DNA-induced upregulation of TNF-α in cultured macrophages and mice serum occurs with the repression of p38 mitogen-activated protein kinase (MAPK) and NF-kB signaling cascades [32]. NF-κB pathway may be directly inhibited by some cyclopentenone-containing prostaglandins with electrophilic properties such as 15-deoxy-D12,15-prostaglandin J_2_ (15d-PGJ_2_), which covalently modify cysteine residue on IKKβ kinase, causing its inactivation [88]. Furthermore, OxPLs attenuate LPS-induced JNK phosphorylation and NF-κB nuclear translocation, thereby mitigating inflammatory responses in macrophages [89]. Another major mechanism involved in OxPL-induced inhibition of inflammation is via increase in intracellular cyclic adenosine monophosphate (cAMP) levels. cAMP-mediated protein kinase A (PKA) pathway is well known for mediating anti-inflammatory effects on EC as exemplified by inhibition of E-selectin [90] and repression of NF-κB activation and ICAM-1 expression [91]. In this regard, various studies have shown that OxPL-stimulated elevation in cAMP levels and associated downstream signaling pathways neutralize inflammatory responses [92,93]. OxPLs induce expression of heme oxygenase-1 (HO-1), an antioxidant enzyme with proven anti-inflammatory activities [21,93,94]. HO-1-mediated catabolism of heme produces carbon monoxide which then represses the production of inflammatory cytokines including IL-1β and TNF-α [95,96]. OxPLs also enhance the expression of other antioxidant enzyme cyclooxygenase-2 (COX-2) in PPARγ- and cAMP response element binding protein (CREB)-dependent manner which exerts inflammatory effects [97]. OxPLs-induced increase in eNOS expression and nitric oxide production can also antagonize inflammatory responses since NO is known to inhibit the expression of EC adhesion molecules and inflammatory cytokine [27]. Pathobiological properties of truncated and full-length OxPL species are summarized in Figure 2.

The activation of redox-responsive transcription factor nuclear factor E2-related factor 2 (Nrf2) serves as an additional mechanism by which OxPLs can mitigate inflammation. Epoxycyclopentenone-containing prostaglandins such as 15d-PGJ2 can bind to Keap1, disrupting the interaction between Keap1-Nrf2, resulting in the release and nuclear translocation of Nrf2, where it induces the expression of antioxidant response genes [98]. The studies have shown that OxPL-induced Nrf2 activation results in resolution of inflammation [99,100]. It appears that phospholipid oxidation products modulate the immune response to induce the expression of anti-inflammatory and pro-resolving molecules to counterbalance the excessive inflammation. Indeed, these pro-resolving molecules such as lipoxins and resolvins are produced as a part of so-called “lipid program switch” at the later stages of inflammation to overcome the harmful effects of pro-inflammatory molecules generated at the early phases [101]. This phenomenon was best illustrated in one of our recent study where we found that OxPAPC stimulates the generation of lipoxin A4 (LXA4) in cultured EC as well as mice lungs that protects against TNF-α or LPS-induced vascular leak and inflammation [35]. The activation of formyl peptide receptor 2 was involved in mediating the anti-inflammatory effects of OxPAPC-generated LXA4.

A number of research groups including ours have been focusing on developing novel classes of OxPLs that have improved cytoprotective, anti-inflammatory, and in vivo stability properties. To this end, in addition to extensively studying the molecular/cellular mechanisms involved in OxPAPC-derived beneficial effects on vascular endothelium, we generated and tested a phospholipase-resistant synthetic phospholipid containing in the *sn*-2 position of a phospholipid a stable analog of prostaglandin I2, iloprost, with known barrier protective and anti-inflammatory effects. Testing of this novel synthetic phospholipid showed more pronounced and prolonged barrier protective and anti-inflammatory effects on lung endothelium and in animal model of LPS-induced lung injury [102]. Lu et al. synthesized 15d-PGJ2-PC that showed potent antioxidant, antifibrotic and anti-inflammatory effects on macrophages by regulating NF-κB, PPARγ, and Nrf2 signaling pathways [103]. Importantly, both of these compounds were effective in attenuating LPS-induced ALI in mice. Lately, IgM antibody raised against OxPL has been shown to protect against atherosclerosis in mice [104].

## 6. OxPLs in the Positive Regulation of Endothelial Barrier Function

One of the best studied beneficial properties of OxPLs is their positive regulation of endothelial barrier function. Endothelial barrier formed by EC monolayers controls the passage of fluids, macromolecules, and immune cells between the blood and underlying tissue. The disruption of this selective and semipermeable barrier results in uncontrolled passage of harmful substances, leading to the development of edema and organ inflammation [105,106]. Studies from our group revealed that OxPAPC induces sustained enhancement basal endothelial barrier function in lung EC [33]. More importantly, OxPAPC accelerates the recovery of thrombin-induced endothelial permeability. Comprehensive studies from our laboratory have described OxPAPC protection in several models of agonist-induced endothelial barrier dysfunction including LPS, TNF-α, *Staphylococcus aureus*, and mechanical forces [34,35,36]. These studies also showed that endothelial barrier protective effects of OxPLs are restricted to purified full-length oxygenated species or total OxPAPC pool containing majority of full length OxPL species at low concentrations. In contrast, fragmented OxPLs products such as POVPC, lyso-PC, and PGPC cause EC barrier disruption, while the oxidation-resistant DMPC compound lacks biological activities toward EC barrier or inflammation control. These observations demonstrate that only full-length but not truncated OxPLs have a positive impact on EC barrier function. This separation of biological activities among OxPL species explains to some extent an apparent controversy in biological effects of OxPLs reported by different groups in different models.

The studies from our group have identified several interrelated receptor-mediated and intracellular signaling pathways, leading to OxPAPC-induced enhancement, protection, and restoration of endothelial barrier. Among these, activation of Rac/Cdc42, a Rho family of small GTPases, followed by actin cytoskeleton remodeling appears to be the unified mechanism involved in conveying barrier protective OxPLs signals. Endothelial barrier undergoes continuous remodeling in response to various agonists/antagonists, which is regulated by small GTPases that constantly shuttle between GTP-bound active and GDP-bound inactive states [107,108]. OxPAPC-stimulated EC show Rac/Cdc42 activation-dependent peripheral actin rim formation that strengthens endothelial barrier [33]. OxPAPC-induced Rac activation is facilitated by Rac/Cdc42-specific guanine nucleotide exchange factors Tiam1 and betaPIX [109]. The enhanced interactions between adherens junction (AJ), tight junction (TJ), and focal adhesion (FA) proteins following Rac activation are essential for OxPAPC-induced enhancement of endothelial barrier integrity, which has been summarized in our recent review [15]. Briefly, a positive feedback loop developed by the interaction between Rac and p21-activated kinase-mediated phosphorylation of Paxillin [109] and by the interaction between AJ protein β-catenin, VE-cadherin with TJ protein ZO-1, FA protein paxillin, and Rac effector proteins Afadin and IQGAP1 all play crucial roles in mediating OxPAPC-induced positive regulation of endothelial barrier function [110,111,112,113,114]. In consistence with the established notion that activation of Rho mediates endothelial barrier disruption, OxPAPC-induced protection of EC barrier integrity involves activation of p190RhoGAP, a negative regulator of Rho [115]. Notably, our multiple studies have demonstrated that OxPAPC protects against lung vascular leak and inflammation in vivo induced by high tidal volume mechanical ventilation, *Staphylococcus aureus*, and LPS [34,35,36,37]. The decrease in expression of ICAM-1, VCAM-1, and inflammatory cytokines and inhibition of NF-κB activation are some of the mechanisms involved in OxPAPC-mediated protection of vascular leak and inflammation in these murine models of lung injury.

**Figure 3:**
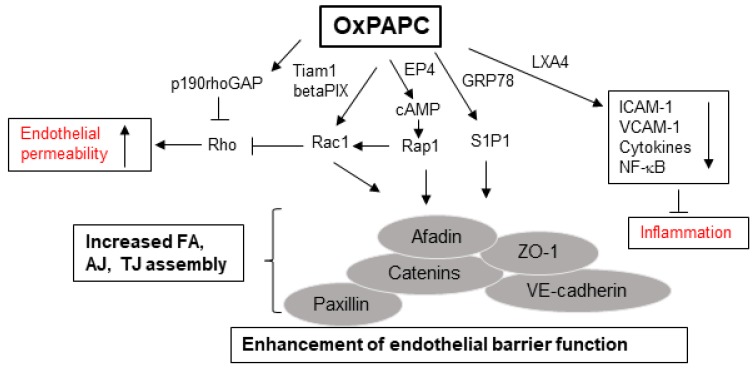
Protective roles of 1-palmitoyl-2-arachidonoyl-sn-glycero-3-phosphorylcholine oxidation (OxPAPC) in endothelial barrier function: OxPAPC augments endothelial cell junctions by increasing the Rac/Rap-mediated interactions of various adherens junctions (AJ), tight junctions (TJ), and focal adhesion (FA) proteins. OxPAPC stimulates the production of cAMP and activates sphingosine 1- phosphate 1 (S1P1) that enhances endothelial barrier integrity. Furthermore, OxPAPC inhibits Rho-mediated increase in endothelial permeability. Simultaneously, OxPAPC represses inflammation by inducing the production of anti-inflammatory molecules such as lipoxin A4 (LXA4).

OxPAPC-stimulated EC exhibits the activation of various kinases including PKC; PKA; Raf-MEK1 and 2; and Erk 1/2 MAPK cascade [116].Furthermore, some receptor-mediated pathways are also critical for OxPAPC-induced EC barrier function. For instance, recruitment of sphingosine 1-phosphate receptor (S1P1) along with Akt, Rac, and Tiam1 to OxPAPC-activated caveolin-enriched microdomains is essential for endothelial barrier enhancement by OxPAPC [117]. S1P1 activation by OxPAPC requires its binding to endoplasmic reticulum- and cell membrane-localized chaperone protein GRP78 [118]. Moreover, a synthetic agonist of S1P1 has been shown to exert potent endothelial barrier protective effects both in vitro and in vivo [119,120]. Interestingly, prostaglandin receptors also seem to mediate barrier-enhancing effects of OxPAPC in EC, as illustrated by one of our recent study where prostaglandin E2 receptor-4 (EP4) was essential for Rac-mediated sustained barrier protective effects of OxPAPC [121]. More importantly, anti-inflammatory effects of OxPAPC were abolished in EP4 knockout mice, highlighting an important role of this prostaglandin receptor in mediating the barrier protective actions of OxPAPC. Signaling pathways mediating permeability and anti-inflammatory effects of OxPAPC are summarized in Figure 3.

## 7. OxPLs in Endothelial Dysfunction

As mentioned above, truncated products of OxPLs induce endothelial barrier disruption and higher doses (50–100 µg/mL) of OxPAPC show similar disruptive effects. Further mechanistic studies deciphered signaling mechanisms underlying these differential physiologic effects. Higher, but not lower, doses of OxPAPC induced excessive ROS production, activation of Src kinase, and phosphorylation of AJ protein VE-cadherin at tyrosine residues 658 and 738. Such VE-cadherin phosphorylation limits its interaction with other AJ proteins, p120-catenin and β-catenin [39]. A similar mechanism is triggered by fragmented OxPLs species, which also induce endothelial permeability via increased ROS production-mediated activation of Src kinase and phosphorylation of VE-cadherin, leading to breakdown of AJ assembly [38]. VE-cadherin is linked to actin cytoskeleton by its interactions with the catenin family of proteins and disassembly of the VE–cadherin–Catenin complex following tyrosine phosphorylation, internalization, or cleavage of VE-cadherin causes the destabilization of AJ with an increase in endothelial permeability [122,123]. In this context, disassembly of AJ complex due to the phosphorylation of VE-cadherin appears to be the unified mechanism of endothelial barrier dysfunction caused by some OxPLs. Further studies suggested a role for vascular endothelial growth factor receptor-2 (VEGFR-2) in the more sustained OxPAPC-induced endothelial barrier disruption caused by higher doses of OxPAPC [124].

## 8. OxPLs in Atherosclerosis

The earliest evidence of pathological roles of OxPLs came from their proven role in the pathogenesis of atherosclerosis, a primary cause of many cardiovascular diseases. There are excellent reviews describing OxPLs contribution to the pathogenesis of cardiovascular disease [8,125], and these works will not be further discussed here. However, it is noteworthy to mention that EC plays a crucial role in OxPL-induced atherosclerotic phenotype. The increased deposition of OxPLs in the arterial wall beneath the endothelium and accompanying excessive EC inflammatory responses have been attributed as two major pathological cascades in the development of atherosclerosis. Accordingly, lowering the levels of OxPLs and inhibiting EC-derived inflammation are being clinically tested for developing therapeutics against OxPL-induced cardiovascular disorders including atherosclerosis. 

## 9. OxPLs in Aging

The significant compromise in the antioxidant defense system with aging makes the aged population more vulnerable to inflammation and oxidative stress-induced pathologies. Since OxPLs are generated via oxidation, the link between redox status of the lung and the levels of truncated, more advance products of phospholipid oxidation, was investigated. Oxidized phopspholipidomics analysis using advance mass-spectrometry approaches revealed that the basal levels of truncated OxPLs—POVPC, PONPC, PGPC, lyso-PC, and PazPC—are higher in the lungs of aged mice (18–24 months) compared to their younger (2–4 months) counterparts [52]. Intratracheal injection of LPS causes higher levels of an accumulation of these truncated OxPLs in aged animals as well as delayed clearance of OxPLs in aged vs young groups. To mimic the compromised defense system of the aged or diseased population, we employed two-hit injury model where sub-pathologic doses of OxPLs were combined with cytokine mixture and where endothelial barrier function and parameters of lung injury were monitored. The low doses of OxPLs which do not cause EC barrier disruption on their own augmented cytokine-induced endothelial dysfunction in a two-hit model. By applying same principle in vivo, we found that truncated OxPLs at minimal doses not affecting endothelial function when added alone lead to exacerbated vascular leak and inflammation when combined with TNF-α. These findings demonstrate that elevated levels of truncated OxPLs are capable of exacerbating ALI in aging population. The generation of truncated OxPLs may be a common mechanism shared by many EC barrier disruptive agonists. For example, exposure of EC to particulate matter (PM) from polluted air triggers production of several fragmented OxPLs [126]. Similar to the augmenting effect on endothelial dysfunction caused by LPS and inflammatory cytokines, low doses of truncated OxPLs combined with low dose of PM that normally does not affect EC barrier properties resulted in profound endothelial barrier dysfunction. Altogether, these observations indicate that even low concentrations of OxPLs may have deleterious effects on the population with compromised antioxidant or immune defense system. Notably, overexpression of platelet-activating factor acetyl hydrolase 2 (PAFAH2), an enzyme specifically hydrolyzing truncated OxPLs, rescued cytokine-induced endothelial dysfunction while pharmacological inhibition of PAFAH2 exacerbated cytokine-mediated inflammation in EC cultures and in mice lungs [52,126]. These observations strongly suggest that removal of truncated OxPLs could be a promising approach to mitigate OxPLs-induced pathologies. 

In agreement with our findings, a study by Liu et al. reported the presence of higher levels of bioactive truncated OxPLs in the plasma of aged mice [127]. An elevated level of fragmented phosphatidylcholine has also been reported in human blood plasma [128]. Another study had shown the increasing concentration of OxPL precursor, phosphatidylcholine, with age in mice [129]. Interestingly, an increase in phosphatidylcholines containing longer chain fatty acids with unsaturated bonds and a reduction in sphingomyelin concentrations was also verified by a recent study showing higher serum levels of ester-linked phosphatidylcholine and phosphatidylethanolamine in older human population compared to younger counterparts [130]. Collectively, these observations suggest that altered lipid metabolism with subsequent changes in bioactive lipid profiles is directly associated with aging. had been found in patients with Alzheimer’s disease [131,132]. An altered lipid profile during aging 

As a major site of ROS generation, mitochondrial lipids may be vulnerable to increased oxidative damage with aging. Cardiolipin, a glycerophospholipid present in the inner membrane of mitochondrial lipid bilayer, is a particular target of oxidation that leads to altered mitochondrial morphology and promotes cell death [133,134]. The oxidation of cardiolipin has been shown to contribute to hyperoxic lung injury [135], and total cardiolipin content has been shown to decrease in mitochondria with an increase in its oxidized forms in hearts and brains from aged rats [136,137]. More recently, a significant reduction in cardiolipins levels in various organs with the most severe effects in aged mice kidney has been reported [138]. These findings suggest towards an important role of aging-associated cardiolipin oxidation in mitochondrial dysfunction associated with various diseases.

## 10. Phospholipidomics: A New Era of Structural-Functional Analysis of OxPLs

With the development of high mass accuracy liquid chromatography-mass spectrometry (LC-MS) equipment and tremendous advancements in bioinformatics, it is now possible to detect and analyze very low-abundance OxPLs species. These developments have enabled to quantify a small fraction of change in oxidation status of various phospholipids and to study signaling pathways associated with their biological function. This next generation technology-assisted analysis will eventually help explain the variability among different studies in the past, reporting the contrasting roles of OxPLs. A recent multi-laboratory LC-MS analysis demonstrated that, although overall oxidation pattern and most abundant OxPLs remained consistent among air-oxidized PAPC prepared and utilized by four laboratories, there was a notable difference in the degree of oxidation [139]. This important comparison clearly suggests that OxPLs from different preparations may not always have identical biological activities. By employing hydrophilic liquid chromatography coupled to MS, Colombo et al. showed that phospholipidomic profile significantly varies among hydrogen peroxide, glucose, or hydroxynonenal-induced stress conditions in bovine aortic EC [140]. Lipidomic analysis can be utilized to determine the pathologic states and thus can be used as a biomarker platform, as exemplified in a study showing the disease-specific differential lipid profiles [141]. Similarly, oxidative phospholipidomics analysis of lipids have identified oxygenated cardiolipins and phosphatidylethanolamines as predictive biomarkers of apoptotic and ferroptic cell death, respectively [142].

## 11. Future Perspectives

**Figure 4:**
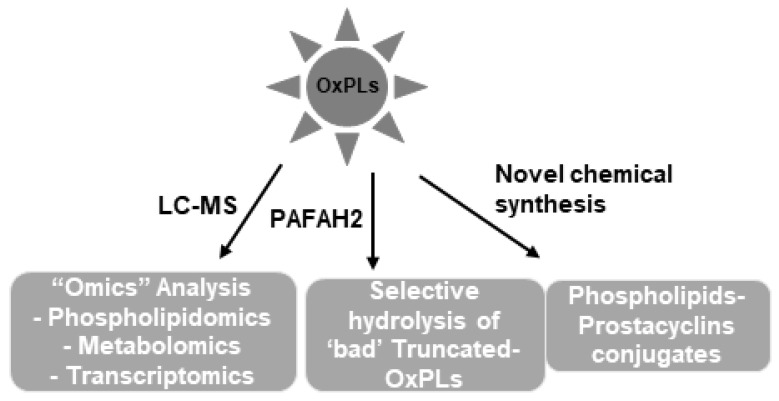
Future directions in OxPLs research: With the development of more advanced LC-MS technologies, precise pathophysiological roles of OxPLs will be determined by various “omics” analyses. In addition, selective removal of harmful OxPLs species by specific enzymes and novel chemical modifications to create new phospholipids molecules with improved functions should also be considered for future researches.

We are only at the beginning of understanding the complex biological nature of heterogenous mixtures of OxPLs generated in the human body. At least, it is now accepted that these OxPLs are not simple lipid peroxidation products; rather, they exhibit both beneficial and deleterious biological effects. The better knowledge on how varieties of OxPLs are produced from the oxidation of a single phospholipid molecule and biological characterization of such species will help define their precise roles in health and disease. Future studies employing advanced LC-MS techniques combined with “omics” (lipidomic, metabolomic, transcriptomic, proteomic, etc.) analysis will provide insights into the role of OxPL-derived cellular signaling in the pathogenesis as well as treatment of lipid-associated diseases such as atherosclerosis, thrombosis, ALI, diabetes, cancers, and others. The latest findings have revealed previously unrecognized roles of OxPLs such as a possible contributing factor in the development of aging-related lung injuries. These findings leave an open question on whether elevation of levels of “bad” OxPLs acts as a critical exacerbating factor in the vulnerable population with existing diseases. The pharmacological/molecular tools such as use of PAFAH2 to selective remove the pathogenic species of OxPLs seem to be an exciting perspective to deal with lipid-induced diseases (Figure 4).

The most significant advancements achieved in OxPL research to date is identification of the anti-inflammatory and endothelial barrier protective properties of OxPAPC. The engagement of multiple receptors and signaling cascades in mediating the beneficial effects of OxPAPC suggest a necessity of combined pharmacological modulation of several molecular targets that may lead to the most efficient therapeutic treatment of a spectrum of conditions associated with pathological effects of truncated OxPLs. On the other hand, the design and preclinical testing of more stable synthetic phospholipid single molecules with controlled composition presents a novel way of engineering more potent and effective therapeutics. This direction has been supported by our study demonstrating superior protective effects of synthetic iloprost-PC compound over free iloprost against inflammatory agonist-induced pulmonary dysfunction [102] (Figure 4). Furthermore, OxPAPC-dependent generation of lipoxin or its analogs [35,99] encourages a future analysis of OxPAPC-derived molecules that might be produced by OxPAPC-exposed vascular endothelium in vivo and might play an important role in the resolution of inflammation.

## 12. Summary

The overlapping signaling pathways and structural heterogeneity rule the dual biological effects of OxPLs. The potent anti-inflammatory and vascular barrier protective effects of specific groups of OxPLs have placed them at the center stage for the development of effective therapeutics against disorders associated with endothelial dysfunction. Utilization of novel molecular approaches to neutralize harmful OxPL species might be an alternative yet complementary strategy to prevent and treat OxPLs-induced pathologies. Future studies are warranted to test the efficacy of already identified molecular targets, to search for additional targets, and to map them to exact body locations by utilizing currently available advanced tools such as phospholipidomics MS quantitation and phospholipidomics imaging.
